# Contribution of “Genuine Microglia” to Alzheimer's Disease Pathology

**DOI:** 10.3389/fnagi.2022.815307

**Published:** 2022-03-24

**Authors:** Sadayuki Hashioka, Ken Inoue, Koji Otsuki, Maiko Hayashida, Rei Wake, Noriyuki Kawano, Haruo Takeshita, Masatoshi Inagaki

**Affiliations:** ^1^Department of Psychiatry, Faculty of Medicine, Shimane University, Izumo, Japan; ^2^Medical Sciences Cluster, Health Service Center, Research and Education Faculty, Kochi University, Kochi, Japan; ^3^The Center for Peace, Hiroshima University, Hiroshima, Japan; ^4^Department of Legal Medicine, Faculty of Medicine, Shimane University, Izumo, Japan

**Keywords:** microglia, monocytes, CNS-associated macrophages (CAMs), transmembrane protein 119 (TMEM119), P2Y purinergic receptor 12 (P2RY12), single-cell profiling, Alzheimer's disease

## Heterogeneity of Myeloid Cells in the Brain

In the late 1980's, McGeer et al. observed that major histocompatibility complex (MHC) class II-immunopositive cells with an amoeboid shape were concentrated in the vicinity or center of amyloid plaques in postmortem brains of patients with Alzheimer's disease (AD) (McGeer et al., 1987; Itagaki et al., 1988). This historical neuropathological finding was interpreted to mean that microglia were activated in lesioned areas, and led to the neuroinflammation hypothesis suggesting that activated microglia significantly contribute to AD pathogenesis. Recently, genome-wide association studies have identified variants of the myeloid cell genes triggering receptor expressed on myeloid cell 2 (*TREM2*) (Jonsson et al., 2013), complement receptor 1 (Lambert et al., 2009), and *CD33* (Hollingworth et al., 2011) as novel AD risk genes, sparking renewed interest in microglia from the aspect of genetics (Hashioka et al., 2020).

However, microglia are not the only myeloid cells expressing MHC class II in the brain. Besides parenchymal microglia, the intact brain hosts non-parenchymal specialized myeloid cells such as perivascular, meningeal, and choroid-plexus macrophages, which are referred to as CNS-associated macrophages (CAMs) (Kierdorf et al., 2019). In addition, circulating monocytes are believed to infiltrate the brain and differentiate into macrophages under pathological conditions (Martin et al., 2017). Human microglia, CAMs, and infiltrating monocytes/macrophages express MHC class II as well as certain pan-macrophage markers, such as Iba1 (ionized calcium-binding adapter molecule 1), CD11b, and the fractalkine receptor CX3CR1 (Prinz et al., 2017; Bottcher et al., 2019; Kierdorf et al., 2019). Identification of these brain mononuclear phagocytes was based on their location, morphology, and a small set of surface markers. Such mononuclear cells, therefore, used to be mingled in conventional bulk analyses.

Accumulating evidence indicates that ontogeny and longevity are prominent properties shared by microglia and CAMs, but not by infiltrating monocytes/macrophages (Prinz et al., 2017; Kierdorf et al., 2019). Microglia and CAMs, excluding choroid-plexus macrophages, arise solely from erythromyeloid progenitor cells in the extraembryonic yolk sac and possess extreme longevity and self-renewal potential, without replacement by circulating monocytes (Ginhoux et al., 2010; Goldmann et al., 2016). As an exception for CAMs, choroid-plexus macrophages show mixed ontogeny and a substantial contribution from circulating monocytes (Goldmann et al., 2016). Immigrating monocytes/macrophages, which express a unique monocytic marker Ly6C in mice (Geissmann et al., 2003), originate from the myeloid progenitor lineage in the bone marrow and exhibit a short life with high turnover (Prinz et al., 2017).

## Specific Markers Segregating Myeloid Cells in the Brain

Although microglia and most CAM populations share the same prenatal origin (i.e., yolk sac), recent studies with single-cell RNA sequencing have clearly segregated the transcriptome signature specific for microglia from that for CAMs. Specifically, *TMEM119* (transmembrane protein 119) and *P2RY12* (P2Y purinergic receptor 12) have been identified as core genes specific to microglia in humans (Masuda et al., 2019; Sankowski et al., 2019), while *Mrc1* (mannose receptor 1, also called CD206) and *Pf4* (platelet factor 4) have been considered as core genes specific to CAMs in mice (Zeisel et al., 2015; Jordao et al., 2019) (Table 1). Indeed, selective microglial expression of TMEM119 and P2RY12 has been confirmed at the protein level in humans. Immunohistochemical analysis of postmortem human brains showed that parenchymal Iba1-immunopositive cells expressed TMEM119 (Bennett et al., 2016; Satoh et al., 2016). On the other hand, the Iba1^+^ or CD68^+^ cells, which were presumed to be infiltrating monocytes, did not express TMEM119 in active demyelinating lesions of multiple sclerosis (MS) or necrotic lesions of cerebral infarction (Satoh et al., 2016). P2RY12 immunoreactivity was also observed in parenchymal Iba1^+^ ramified cells that were supposed to be microglia, but not in CD14^+^ and CD16^+^ cells in blood vessels and in the meninges. These cells most likely correspond to peripherally derived monocytes and meningeal macrophages, respectively (Mildner et al., 2017). Postmortem brain study demonstrated that such microglial expression of P2RY12 was decreased in the brains of AD patients and those of MS patients (Mildner et al., 2017). More critically, another advanced single-cell technology, namely cytometry by time of flight (CyTOF), showed that TMEM119 and P2RY12 were expressed on microglia isolated from postmortem human brains and were absent from myeloid cells in human blood and cerebrospinal fluid (Bottcher et al., 2019). Accordingly, in humans, it is tempting to regard TMEM119 and P2RY12 as the most reliable markers that can identify “genuine microglia” in humans, while *Siglech* (sialic-acid-binding immunoglobulin-like lectin-h) (Bedard et al., 2007; Konishi et al., 2017) and *Hexb* (beta-hexosaminidase subunit beta) (Masuda et al., 2020; Jia et al., 2021) are also considered as microglia-enriched genes (Table 1).

**Table 1:** Markers of myeloid cells in the brain.

**Marker**	**Target Cell Type**	**Origin of Target Cell**	**References**
TMEM119	Homeostatic microglia	Yolk sac	Bennett et al., 2016; Bottcher et al., 2019; Masuda et al., 2019
P2RY12	Homeostatic microglia	Yolk sac	Mildner et al., 2017; Bottcher et al., 2019; Sankowski et al., 2019
Siglech	Homeostatic microglia	Yolk sac	Bedard et al., 2007; Konishi et al., 2017
Hexb	Microglia during homeostasis and disease	Yolk sac	Masuda et al., 2020; Jia et al., 2021
Clec7a	DAM/MGnD	Yolk sac	Keren-Shaul et al., 2017; Krasemann et al., 2017
Mrc1 (CD206)	CAMs (except for some choroid-plexus macrophages)	Yolk sac	Zeisel et al., 2015; Jordao et al., 2019
Pf4	CAMs (except for some choroid-plexus macrophages)	Yolk sac	Jordao et al., 2019
Ly6C	Infiltrating monocytes/macrophages	Bone marrow	Geissmann et al., 2003

*DAM, disease-associated microglia; MGnD, microglial neurodegenerative phenotype; CAMs, CNS-associated macrophages*.

## Pathological Roles of Heterogenous Brain Myeloid Cells in Alzheimer's Disease

The heterogeneous nature of brain myeloid cells raises the question as to whether or not there are differences in pathological roles between microglia, CAMs, and infiltering monocytes in AD. Do “genuine microglia” play specific roles in AD pathogenesis? The answer seems to be yes, since it was demonstrated that macrophages transplanted from the bone marrow in donors could adopt some features of endogenous microglia, but such macrophages were not able to fully recapitulate all microglial properties, such as increased expression of microglial identity genes, even after prolonged residence in the recipient brain (Bennett et al., 2018).

In addition, several studies have reported conflicting results concerning the contribution of microglia, CAMs, and recruited monocytes to AD pathology. For instance, infusion of wildtype monocytes derived from the bone marrow to the peripheral blood of AD transgenic mice led to spontaneous migration of monocytes to amyloid lesions in the absence of irradiation, genetic manipulation, or chemotherapy. Such treated mice showed a decrease in cerebral Aβ levels, which seemed to be associated with monocytic phagocytosis, and ameliorated cognitive deficits (Koronyo et al., 2015). On the other hand, a study using AD transgenic mice demonstrated that peripheral monocytes distinguished from microglia by parabiosis were not significantly recruited to Aβ plaques, whereas resident microglia gathered to surround Aβ plaques (Wang et al., 2016). This controversy may stem from limitations of conventional analytical methods, such as immunohistochemistry and flow cytometry, employed in the aforementioned studies to characterize myeloid cells. These methods can only probe a few preselected proteins as cell surface markers.

But now, can “genuine microglia” reliably be typified by the microglia-specific markers TMEM119 and P2RY12, which were established by the latest single-cell profiling technologies? There seems to be no clear answer, since expression levels of TMEM119 and P2RY12 depend on the microglial activation status. Microglia highly express microglia core genes *TMEM119* and *P2RY12* in the homeostatic state. After loss of their homeostatic phenotype, however, microglia suppress the expression of *TMEM119* and *P2RY12* in an activated state referred to as disease-associated microglia (DAM) (Keren-Shaul et al., 2017) or microglial neurodegenerative phenotype (MGnD) (Krasemann et al., 2017). Such DAM/MGnD microglia are closely associated with Aβ plaques and possess pro-inflammatory signatures (Keren-Shaul et al., 2017; Krasemann et al., 2017). Based on these findings, TMEM119 and P2RY12 should be regarded as homeostatic microglia molecules. Therefore, immunohistochemical analysis appears to be difficult to distinguish between the absence of microglia themselves and the presence of microglia in the DAM/MGnD activation state in lesions showing poverty of sole TMEM119 or P2RY12 immunoreactivity.

A recent study using immunocytochemical electron microscopy has uncovered a new microglial phenotype called dark microglia, which are associated with amyloid plaques in APP/PS1 mice (Bisht et al., 2016). Dark microglia display condensed, electron-dense cytoplasm and nucleoplasm, a characteristic giving them a “dark” appearance. They also show a downregulated expression of the homeostatic marker P2RY12 (Bisht et al., 2016). Therefore, gene expression profiles and biological characteristics of dark microglia may be similar to those of the DAM/MGnD microglia.

## Novel Genetic Approaches to Define Pathological Roles of Microglia

Advances in genetic manipulation have established certain transgenic or knock-in mouse lines that genetically target microglia. Such mouse lines have been shown to monitor precisely and manipulate microglia regardless of their activation state. In addition, CyTOF combined with fate mapping on APP/PS1 mice has detected a subset of microglia associated with AD-prone neurodegeneration (Mrdjen et al., 2018).

The mice with the recombinase *CreERT2* inserted into the locus *TMEM119* and the *TMEM119*-*tdTomato* knock-in mice have shown clear discrimination of microglia from CAMs, even though non-myeloid brain cells, such as endothelial cells and fibroblasts, and some choroid-plexus macrophages can also be targeted (Kaiser and Feng, 2019; Ruan et al., 2020). In the brains of *Tmem119*-*tdTomato* reporter mice that were treated by laser ablation, tdTomato-positive microglia, which were presumably activated, entered the site of injury and dramatically changed their process length without losing the TMEM119-tdTomato signal (Ruan et al., 2020). It is yet to be clarified why TMEM119 expression was preserved even in microglia activated in lesioned areas. Also, *P2RY12-CreERT2* knock-in mice were able to specifically label microglia, as shown by *P2ry12*-*CreERT2*; *Rosa26*^*Rosa*26*Ai*14^ reporter mice that expressed TdTomato upon Cre-dependent recombination with minor effects on CAMs (McKinsey et al., 2020). When *P2RY12*-*CreERT2*; *Rosa26*^*Rosa*26*Ai*14^ mice underwent middle cerebral artery occlusion, tdTomato^+^ microglia exhibited reduced immunoreactivity for P2RY12 and TMEM119 in the ischemic core and penumbra (McKinsey et al., 2020).

To define the pathological roles of “genuine microglia” in AD, it is tempting to apply these mouse lines targeting microglia genetically to experimental AD models. For instance, intrahippocampal injection of Aβ into such mice seems to be technically feasible and to facilitate the transcriptional and functional analysis of microglia in response to Aβ. It should be noted that there is no AD animal model sufficient to reflect all aspects of AD pathology (Drummond and Wisniewski, 2017). In fact, intrahippocampal Aβ injection appears to exaggerate inflammatory responses and to represent an acute brain insult (McLarnon and Ryu, 2008), even though chronic inflammation is considered a critical aspect of AD pathology. Nevertheless, further studies along this line are warranted to elucidate the potential of microglia as key therapeutic targets in AD.

Tracing DAM/MGnD microglia in the brain of AD animal models could also help to reveal the pathological roles of microglia. DAM/MGnD microglia have been identified by high expression of the DAM/MGnD marker Clec7a combined with low expression of the homeostatic microglia marker P2RY12 (Keren-Shaul et al., 2017; Krasemann et al., 2017). While clarifying microglial ontogeny in humans could help to clarify the pathological roles of human microglia, there is no approach to address this issue directly for obvious reasons. However, using single-cell transcriptional profiling approaches, a recent study on macrophages from aborted fetuses implies that human microglia are derived largely from yolk sac progenitors (Bian et al., 2020).

## Author Contributions

SH wrote the manuscript. All authors discussed, edited the manuscript, read, and approved the final manuscript.

## Funding

This study was supported by JSPS KAKENHI Grant Numbers 19K08018 (SH) and 19H04355 (SH and NK).

## Conflict of Interest

The authors declare that the research was conducted in the absence of any commercial or financial relationships that could be construed as a potential conflict of interest.

## Publisher's Note

All claims expressed in this article are solely those of the authors and do not necessarily represent those of their affiliated organizations, or those of the publisher, the editors and the reviewers. Any product that may be evaluated in this article, or claim that may be made by its manufacturer, is not guaranteed or endorsed by the publisher.
